# The factors that influence the oral health-related quality of life in 12-year-old children: baseline study of a longitudinal research

**DOI:** 10.1186/s12955-017-0729-2

**Published:** 2017-08-07

**Authors:** Ling Sun, Hai Ming Wong, Colman P. J. McGrath

**Affiliations:** 1 Paediatric Dentistry & Orthodontics, Faculty of Dentistry, The University of Hong Kong, 2/F, Prince Philip Dental Hospital, 34 Hospital Road, Sai Ying Pun, Hong Kong; 2 Periodontology and Public Health, Faculty of Dentistry, The University of Hong Kong, Sai Ying Pun, Hong Kong

**Keywords:** Oral health-related quality of life, Periodontal status, Caries, Malocclusion, Sociodemographic factors, Baseline study

## Abstract

**Background:**

Oral health-related quality of life (OHRQoL) could be affected not only by oral health but also by demographic and ecosocial factors. This research aimed to analyze the sociodemographic and clinical factors that may influence the OHRQoL of 12-year-old children.

**Methods:**

A representative sample was selected from Hong Kong. Periodontal status and caries were examined according to WHO criteria. Four orthodontic indices were used to assess malocclusion. Child Perception Questionnaires (CPQ_11–14_-ISF:8 and CPQ_11–14_-RSF:8) including four domains, namely oral symptoms (OS), functional limitations (FL), emotional well-being (EWB), and social well-being (SWB), were used to measure OHRQoL. Adjusted OR was calculated by ordinal logistic regression.

**Results:**

Totally 589 eligible subjects (305 females, 284 males) were recruited. Males tended to rank higher in OS domain but lower in EWB domain (adjusted OR = 1.89 and 0.67). Mother’s education was linked more closely with children’s CPQ scores. Higher education levels were associated with better quality of life (adjusted OR = 0.45 and 0.37). Household income showed no effect on CPQ scores. Unhealthy periodontal conditions had a negative effect on EWB and total CPQ (adjusted OR = 1.61 and 1.63). High caries experience only had a negative effect on SWB (adjusted OR = 1.60). Malocclusion affected FL, EWB, SWB and total CPQ: all malocclusion severities affected SWB; only severe malocclusions affected FL, EWB and total CPQ.

**Conclusion:**

Males were more tolerant of oral symptoms than females were. Higher levels of mother’s education led to better OHRQoL of their children. Unhealthy periodontal conditions affected emotional well-being, while high caries experience affected social well-being. All malocclusion severities had an effect on social well-being; severe malocclusion further caused functional limitations, worse emotional well-being, and hence worse OHRQoL.

## Background

Clinical techniques in dentistry have been developed rapidly. The aim of these techniques is to give subjects a better life experience. Thus the psychosocial aspects of dentistry have also been researched extensively. Contemporarily researchers are focusing on dental fear, treatment expectations and oral health-related quality of life (OHRQoL). A hypothesis is that subjects’ OHRQoL is affected not only by oral health status but also by other demographic and ecosocial factors. Subjects of different ages, with different education levels and financial situations may put different emphases on their dental care. Those with limited concern of oral health protection may suffer more from dental diseases, hence worse OHRQoL.

Although many studies were conducted on the influence factors of OHRQoL, a consensus has not been reached. This is mainly because different studies included different sampling methods, age groups and influence factors. In addition, most studies adopted cross-sectional design; many articles have recommended that population-based longitudinal study is helpful in this area [1–7]. This article is a baseline study of a longitudinal research aiming to analyze the impact factors of OHRQoL. The cohort of this study was comprised of 12-year-old students in Hong Kong. The subjects will be further studied in their 15- and 18- years old.

## Methods

### Measurement instruments

Different measurement tools can assess OHRQoL. For children aged 11 to 14 years old, the questionnaire of Child Perception Questionnaire (CPQ_11–14_) has been widely validated and used [8–12]. The questionnaire consists of four domains namely oral symptoms domain (OS, 6 items), functional limitations domain (FL, 9 items), emotional well-being domain (EWB, 9 items) and social well-being domain (SWB, 13 items). Each item has a 5-point response format ranging from 0 to 4. The item scores of each domain are added together to get a domain score, and four domains scores are added together to get the total CPQ_11–14_ score. Higher scores represent poorer quality of life.

To facilitate its use in clinical settings and population-based surveys, CPQ_11–14_ was shortened to 16 and 8 items by item impact and stepwise regression methods [13]. Previous studies concluded that the short forms of CPQ_11–14_ (ISF:8 and RSF:8) contained sufficient information in measuring OHRQoL of children in Hong Kong; they were shown to be valid and reliable [14, 15]. The short forms of CPQ_11–14_ (ISF:8 and RSF:8) were used in this research.

Community Periodontal Index (CPI) and the Decayed, Missing and Filled Teeth (DMFT) were used to measure periodontal and caries conditions according to the criteria of WHO [16]. Also, Significant Caries Index (SiC index) was used to classify caries. Individuals are sorted according to their DMFT values; the one third of the population with the highest caries score is selected and the mean DMFT for this subgroup is calculated; this value constitutes the SiC Index [17].

Index of Orthodontic Treatment Need (IOTN), Dental Aesthetic Index (DAI), Index of complexity, outcome and need (ICON), and peer assessment rating (PAR) were used to assess orthodontic treatment need and complexity [18–23].

IOTN was introduced form the UK in 1989, which includes two components of Dental health component (DHC) and Aesthetic component (AC). DHC is originated from the Index of the Swedish Medical Health Board [24]. It has 5 grades (no need to very great need) and the worst occlusal trait is recorded to allocate the grade. AC is comprised of 10 front view photographs selected by non-dental judges from 1000 photographs of 12-year-old subjects, which representing the 10 scales of dental attractiveness. The IOTN (DHC) or IOTN (AC) grading can be further categorized into three orthodontic treatment groups (DHC 1–2 or AC 1–4, no need; DHC 3 or AC 5–7, borderline need; DHC 4–5 or AC 8–10, definite need) [25, 26].

The index of DAI was created in 1986 from the United States. The index was based on approximately 2000 adolescents and adults’ perceptions on the aesthetics of 200 photographs of occlusal configurations. These 200 occlusal configurations were randomly selected from 1337 study models of 15–18 years age [23]. It used regression analysis to choose 10 occlusal traits and put weights on them. The malocclusion measurements are multiplied by their weights, the addition of their products and the addition of a constant number, 13, is the final DAI score. It can be categorized into 4 scales of orthodontic severity and treatment need (<=25, normal or minor malocclusion-no treatment need or slight need; 26–30, definite malocclusion-treatment selective; 31–35: severe malocclusion-treatment highly desirable; > = 36: very severe (handicapping) malocclusion-treatment mandatory) [22]. DAI has been adopted by WHO to examine malocclusion in oral health surveys [16].

ICON was introduced from the UK in 2000 to evaluate treatment need, treatment outcome and complexity [20]. It was based on 97 international orthodontists’ opinion on 240 dental casts for treatment need, and 98 pairs of pre- and post-treatment casts for treatment outcomes. The aesthetic score is assessed using IOTN (AC). Five malocclusion traits are assigned with different weights by stepwise multiple logistic regression. These occlusal trait scores are then multiplied by their respective weightings and summed to calculate the ICON score. The ICON score can be scaled into 2 categories for treatment need (<=43 No; >43 Yes), and 5 categories for orthodontic complexity (<29 easy; 29–50, mild; 51–63 moderate; 64–77 difficult; >77 very difficult). It puts heavy emphasis on aesthetics.

PAR was introduced from the UK based on 10 experts’ estimate of over 200 dental casts. The dental casts represented development as well as pre- and post-treatment stages. The concept is to assign a score to 11 components of occlusal traits that make up a malocclusion. The individual scores are summed together to obtain an overall total, representing the degree a case deviates from normal occlusion. Generally a measure of 10 or less indicates an acceptable alignment and occlusion, and 5 or less suggests an almost ideal occlusion [21].

### Study population and data collection

Cluster randomized trial was used in this research. The sampling frame was all local secondary schools in Hong Kong (by law all children are required to attend secondary school). A random sample of 45 schools (approximately 10% of all local secondary schools) from 18 districts in Hong Kong, SAR, was selected. Students born between April 1st and May 31st, 1997 were invited to participate in oral health survey in 2010 conducted by Faculty of Dentistry, the University of Hong Kong. The sample of this study was selected from the birth cohort of “children of 1997” [27]. Sample size was calculated based on a previous study [28–30], with the prevalence of orthodontic treatment need (ICON) being 80.3%, and the mean total CPQ score (SD) being no need: 14.8 (15.0), and need 20.1 (14.0). With α = 0.05, 1-β = 0.8, design effect for cluster sampling, and a lost rate of 30% at each follow-up considered, the sample sizes at ages 12, 15, and 18 should be 237, 166, and 116, respectively.

An invitation letter was first sent to the parents/primary caregivers. If a written consent from parents/primary caregivers and a verbal consent from students were obtained, students’ oral health status would be examined using an intra-oral disposable mouth mirror with a built-in LED light source. The same trained and calibrated examiner performed the oral examination according to the criteria of WHO [16]. Front-view dental photos were taken by extracting lips using oral retractors to assess IOTN (AC). Dental impressions were collected and the plaster models were sent to OrthoLab (Poland) to make digital models. Software O3DM (version3.8.5 (c) by OrthoLab, Poland) was used to analyze digital models by the same examiner. Reassessments were performed among 10% randomly selected samples after 2 weeks of first assessment to test intra-examiner’s reliability.

Systematic health information, dental treatment history, ecosocial factors including father’s education, mother’s education, and household income were collected from a self-completed questionnaire. OHRQoL was assessed by inviting participants to answer questions of CPQ_11–14_-ISF:8 and CPQ_11–14_-RSF:8.

Subjects were excluded from the final analysis if they were systemically unhealthy, had orthodontic treatment history, or had oral diseases other than caries, periodontitis and malocclusion. Missing data in questionnaires was filled with the mode of the corresponding category.

### Ethics, consent and permissions

The ethical approval of this study was granted by the Institutional Review Board of the University of Hong Kong/Hospital Authority Hong Kong West Cluster (UW 09–453).

### Statistical methods

Intra-examiner reliability was tested by kappa values for CPI, weighted kappa for IOTN (DHC) and IOTN (AC), and Intra-class correlation coefficient (ICC) for DAI score, ICON score and DMFT.

Mann-Whitney U test was used to analyze whether there was a difference of oral health status between females and males; independent samples t test was used to detect the difference of mean DMFT.

The effects of sociodemographic and clinical factors on OHRQoL were analyzed with parameters set as follows:

1. Dependent variables: for bivariate analysis, dependent variables were set as the scores of OS, FL, EWB, SWB and total CPQ; for ordinal regression, dependent variables were set by grouping these scores into four ranks with quartile values as cut-off points.
2. Independent variables: gender, father’s education, mother’s education, household incoming, periodontal status, caries experience, and orthodontic treatment need.
3. Bivariate analysis: for parametric tests, comparison between two samples used the independent samples t test, others used the one-way ANOVA; for nonparametric tests, comparison between two samples used the Mann-Whitney U test, others used the Kruskal-Wallis H test.
4. Multivariate analysis: ordinal logistic regression was used to calculate adjusted odds ratios (OR). To avoid interaction effect, orthodontic treatment needs measured by different orthodontic indices were entered into regression separately.

## Results

Totally 668 students participated in this research in 2010, of whom 589 were eligible for the final analysis (305 females, 284 males). Kappa value for CPI was 0.740; weighted kappa for IOTN (DHC) and IOTN (AC) were 0.918 and 0.790; ICC for DAI score, ICON score and DMFT were 0.821, 0.820 and 0.990.

Missing data only existed in some questions of family information and CPQ_11–14._ Totally 25 subjects had missing data of one or two questions, which were filled with the mode of the corresponding questions.

In this 12-year-old cohort, no differences of oral health status were found between females and males (Table 1). The mean DMFT (SD) was 0.57 (1.02) and the SiC index value (SD) was 1.68 (1.12). Unhealthy periodontal conditions were much more prevalent than caries (86.4% and 31.6%, respectively). The orthodontic treatment need was 45.5% by IOTN (DHC), 20.4% by IOTN (AC), 47.0% by DAI, 35.0% by ICON, and 36.2% by PAR.

**Table 1:** Profile of 12-year-old participants

	Female		Male		Total		P
	N	Percentage	N	Percentage	N	Percentage	
IOTN (DHC) treatment need							
No need	168	55.1%	153	53.9%	321	54.5%	0.845
Borderline need	53	17.4%	53	18.7%	106	18.0%	
Definite need	84	27.5%	78	27.5%	162	27.5%	
IOTN (AC) treatment need							
No need	249	81.6%	220	77.5%	469	79.6%	0.236
Borderline need	40	13.1%	49	17.3%	89	15.1%	
Definite need	16	5.2%	15	5.3%	31	5.3%	
DAI severity and treatment need							
Normal or minor malocclusion- no treatment need or slight need	169	55.4%	143	50.4%	312	53.0%	0.118
Definite malocclusion- treatment selective	75	24.6%	68	23.9%	143	24.3%	
Severe malocclusion- treatment highly desirable	41	13.4%	46	16.2%	87	14.8%	
Very severe (handicapping) malocclusion-treatment mandatory	20	6.6%	27	9.5%	47	8.0%	
ICON treatment need							
No	201	65.9%	182	64.1%	383	65.0%	0.644
Yes	104	34.1%	102	35.9%	206	35.0%	
ICON complexity							
Easy	87	28.5%	86	30.3%	173	29.4%	0.898
Mild	158	51.8%	134	47.2%	292	49.6%	
Moderate	32	10.5%	35	12.3%	67	11.4%	
Difficult	16	5.2%	17	6.0%	33	5.6%	
Very difficult	12	3.9%	12	4.2%	24	4.1%	
PAR							
Almost ideal occlusion	63	20.7%	59	20.8%	122	20.7%	0.530
Acceptable occlusion	137	44.9%	117	41.2%	254	43.1%	
Malocclusion	105	34.4%	108	38.0%	213	36.2%	
Periodontal status							
CPI score = 0	43	14.1%	37	13.0%	80	13.6%	0.705
CPI score > 0	262	85.9%	247	87.0%	509	86.4%	
Caries experience							
< SiC Index value	254	83.3%	245	86.3%	499	84.7%	0.314
> =SiC Index value	51	16.7%	39	13.7%	90	15.3%	
DMFT = 0	203	66.6%	200	70.4%	403	68.4%	0.314
DMFT > 0	102	33.4%	84	29.6%	186	31.6%	
DMFT		Mean (SD)		Mean (SD)		Mean (SD)	
	305	0.59 (1.01)	284	0.54 (1.04)	589	0.57 (1.02)	0.598

P: comparison for DMFT used the independent samples t test; others used the Mann-Whitney U test

SiC *Index* Significant Caries Index; SiC index value (SD) was 1.68 (1.12)

Results of statistical analysis

For bivariate analysis (Table 2), parameter analyses identified almost the same significant factors with non-parameter analyses. Males had a higher OS score (ISF:8) but a lower EWB score (RSF:8) than females.

**Table 2:** Bivariate analysis between the factors and the CPQ_11–14_ scores

		OS (ISF:8)				FL (ISF:8)				EWB (ISF:8)				SWB (ISF:8)				CPQ_11–14_ total score (ISF:8)				OS (RSF:8)				FL (RSF:8)				EWB (RSF:8)				SWB (RSF:8)				CPQ_11–14_ total score (RSF:8)			
	N	Mean (SD)	Median(IQR)	P1	P2	Mean (SD)	Median (IQR)	P1	P2	Mean (SD)	Median (IQR)	P1	P2	Mean (SD)	Median (IQR)	P1	P2	Mean (SD)	Median (IQR)	P1	P2	Mean (SD)	Median (IQR)	P1	P2	Mean (SD)	Median (IQR)	P1	P2	Mean (SD)	Median (IQR)	P1	P2	Mean (SD)	Median (IQR)	P1	P2	Mean (SD)	Median (IQR)	P1	P2
Gender																																									
F	305	3.26(1.29)	3.00(2)	0.000**	0.000**	1.19(1.25)	1.00(2)	0.148	0.329	1.63(1.50)	2.00(3)	0.110	0.061	1.10(1.24)	1.00(2)	0.842	0.786	7.19(3.56)	7.00(5)	0.279	0.372	2.66(1.39)	3.00(2)	0.108	0.110	1.21(1.20)	1.00(2)	0.232	0.096	1.78(1.44)	2.00(2)	0.026*	0.013*	1.42(1.45)	1.00(2)	0.657	0.995	7.08(3.79)	7.00(5)	0.646	0.550
M	284	3.65(1.37)	4.00(2)			1.35(1.44)	1.00(2)			1.43(1.53)	1.00(2)			1.08(1.25)	1.00(2)			7.51(3.77)	7.00(5)			2.85(1.42)	3.00(2)			1.09(1.27)	1.00(2)			1.51(1.47)	1.00(3)			1.48(1.60)	1.00(3)			6.93(3.92)	7.00(5)		
Total	589	3.45(1.34)	4.00(1)			1.26(1.35)	1.00(2)			1.54(1.51)	1.00(2)			1.10(1.24)	1.00(2)			7.34(3.66)	7.00(5)			2.75(1.41)	3.00(2)			1.15(1.23)	1.00(2)			1.65(1.46)	1.00(3)			1.45(1.52)	1.00(2)			7.01(3.85)	7.00(5)		
Father’s education																																									
Primary school graduate or below	88	3.24(1.47)	3.00(2)	0.016*	0.019*	1.27(1.44)	1.00(2)	0.796	0.969	1.34(1.57)	1.00(2)	0.295	0.200	1.03(1.19)	1.00(2)	0.717	0.889	6.89(3.78)	7.00(5)	0.218	0.177	2.60(1.47)	3.00(2)	0.043*	0.029*	1.33(1.36)	1.00(2)	0.139	0.119	1.48(1.45)	1.00(3)	0.471	0.387	1.35(1.31)	1.00(2)	0.779	0.947	6.76(3.97)	6.50(5)	0.468	0.475
Secondary school graduate or below	406	3.55(1.32)	4.00(1)			1.24(1.29)	1.00(2)			1.60(1.52)	1.00(3)			1.12(1.29)	1.00(2)			7.52(3.64)	7.00(5)			2.85(1.38)	3.00(2)			1.16(1.21)	1.00(2)			1.67(1.47)	2.00(3)			1.46(1.52)	1.00(2)			7.14(3.88)	7.00(6)		
College graduate or above	95	3.19(1.26)	3.00(2)			1.35(1.52)	1.00(2)			1.45(1.44)	1.00(2)			1.03(1.11)	1.00(2)			7.02(3.61)	7.00(6)			2.48(1.42)	2.00(1)			0.97(1.22)	1.00(1)			1.72(1.37)	1.00(2)			1.51(1.73)	1.00(3)			6.67(3.58)	7.00(5)		
Mother’s education																																									
Primary school graduate or below	79	3.61(1.59)	4.00(2)	0.381	0.179	1.49(1.42)	1.00(2)	0.246	0.262	2.00(1.78)	2.00(3)	0.014*	0.049*	1.19(1.20)	1.00(2)	0.644	0.542	8.29(4.24)	8.00(6)	0.044*	0.079	3.13(1.45)	3.00(2)	0.010*	0.003**	1.59(1.59)	1.00(3)	0.002**	0.028*	1.95(1.54)	2.00(2)	0.120	0.117	1.84(1.54)	2.00(2)	0.045*	0.026*	8.51(4.51)	8.00(7)	0.001**	0.005**
Secondary school graduate or below	438	3.44(1.29)	4.00(1)			1.22(1.31)	1.00(2)			1.47(1.48)	1.00(2)			1.09(1.27)	1.00(2)			7.22(3.57)	7.00(4)			2.74(1.41)	3.00(2)			1.10(1.16)	1.00(2)			1.59(1.45)	1.00(3)			1.37(1.50)	1.00(2)			6.80(3.72)	7.00(5)		
College graduate or above	72	3.31(1.35)	3.00(2)			1.29(1.51)	1.00(2)			1.46(1.29)	1.50(2)			1.00(1.15)	1.00(2)			7.06(3.44)	7.00(5)			2.44(1.29)	2.00(1)			0.99(1.14)	1.00(2)			1.69(1.34)	2.00(2)			1.49(1.57)	1.00(3)			6.61(3.49)	7.00(5)		
Household income																																									
Below HK$10,000	145	3.35(1.37)	3.00(2)	0.143	0.058	1.16(1.32)	1.00(2)	0.443	0.314	1.48(1.55)	1.00(2)	0.480	0.625	1.14(1.33)	1.00(2)	0.555	0.778	7.13(3.68)	7.00(6)	0.258	0.216	2.63(1.37)	3.00(2)	0.106	0.086	1.20(1.21)	1.00(2)	0.506	0.501	1.60(1.39)	1.00(3)	0.676	0.795	1.48(1.43)	1.00(2)	0.586	0.353	6.91(4.03)	7.00(6)	0.195	0.214
HK$10,001-HK$20,000	213	3.61(1.32)	4.00(1)			1.24(1.29)	1.00(2)			1.59(1.53)	1.00(3)			1.17(1.33)	1.00(2)			7.61(3.75)	7.00(5)			2.88(1.41)	3.00(2)			1.15(1.21)	1.00(2)			1.70(1.58)	2.00(3)			1.49(1.56)	1.00(2)			7.23(3.75)	7.00(4)		
HK$20,001-HK$30,000	92	3.30(1.37)	3.00(2)			1.26(1.46)	1.00(2)			1.47(1.45)	1.00(2)			1.04(1.09)	1.00(2)			7.08(3.67)	7.00(6)			2.83(1.44)	3.00(2)			1.07(1.26)	1.00(2)			1.67(1.40)	1.50(3)			1.29(1.61)	1.00(2)			6.86(3.93)	7.00(5)		
HK$30,001-HK$40,000	62	3.58(1.31)	4.00(1)			1.55(1.35)	1.00(2)			1.81(1.68)	2.00(3)			1.03(1.16)	1.00(2)			7.97(3.21)	8.00(5)			2.89(1.46)	3.00(2)			1.35(1.39)	1.00(2)			1.77(1.44)	2.00(2)			1.65(1.53)	2.00(3)			7.66(3.97)	7.50(5)		
Over HK$40,001	77	3.26(1.34)	3.00(2)			1.30(1.43)	1.00(2)			1.38(1.35)	1.00(2)			0.91(1.07)	1.00(2)			6.84(3.69)	6.00(6)			2.43(1.34)	2.00(2)			1.01(1.18)	1.00(2)			1.45(1.30)	1.00(3)			1.31(1.48)	1.00(2)			6.21(3.48)	5.00(6)		
Periodontal status																																									
CPI score = 0	80	3.34(1.40)	3.00(2)	0.428	0.172	1.06(1.22)	1.00(2)	0.149	0.174	1.21(1.38)	1.00(2)	0.039*	0.037*	0.84(1.01)	1.00(1)	0.020*	0.073	6.45(3.58)	6.00(4)	0.019*	0.008**	2.68(1.42)	3.00(2)	0.598	0.530	1.09(1.28)	1.00(2)	0.602	0.426	1.40(1.40)	1.00(2)	0.100	0.087	1.34(1.42)	1.00(2)	0.478	0.541	6.50(3.63)	6.00(5)	0.207	0.196
CPI score > 0	509	3.47(1.33)	4.00(1)			1.30(1.37)	1.00(2)			1.59(1.53)	1.00(3)			1.14(1.27)	1.00(2)			7.49(3.66)	7.00(5)			2.76(1.41)	3.00(2)			1.17(1.23)	1.00(2)			1.69(1.46)	2.00(3)			1.47(1.54)	1.00(2)			7.08(3.88)	7.00(5)		
Caries experience																																									
< SiC Index value	499	3.43(1.34)	4.00(1)	0.570	0.713	1.24(1.35)	1.00(2)	0.229	0.125	1.52(1.52)	1.00(2)	0.418	0.297	1.05(1.20)	1.00(2)	0.048*	0.078	7.24(3.66)	7.00(6)	0.098	0.098	2.72(1.41)	3.00(2)	0.213	0.233	1.15(1.26)	1.00(2)	0.774	0.382	1.61(1.44)	1.00(3)	0.143	0.157	1.38(1.51)	1.00(2)	0.014*	0.008**	6.87(3.82)	7.00(5)	0.038*	0.037*
> =SiC Index value	90	3.52(1.35)	4.00(1)			1.42(1.32)	1.00(2)			1.66(1.47)	2.00(3)			1.33(1.44)	1.00(2)			7.93(3.66)	8.00(5)			2.92(1.39)	3.00(2)			1.19(1.08)	1.00(2)			1.86(1.53)	2.00(3)			1.81(1.56)	2.00(3)			7.78(3.92)	7.50(6)		
IOTN (DHC) treatment need																																									
No need	321	3.46(1.31)	4.00(1)	0.513	0.710	1.13(1.26)	1.00(2)	0.024*	0.047*	1.48(1.47)	1.00(2)	0.368	0.273	0.98(1.16)	1.00(2)	0.018*	0.052	7.05(3.46)	7.00(5)	0.078	0.159	2.72(1.38)	3.00(2)	0.783	0.708	1.12(1.18)	1.00(2)	0.617	0.293	1.55(1.36)	1.00(3)	0.200	0.381	1.44(1.53)	1.00(2)	0.482	0.307	6.83(3.63)	7.00(5)	0.335	0.380
Borderline need	106	3.55(1.40)	4.00(1)			1.36(1.36)	1.00(2)			1.50(1.64)	1.00(3)			1.09(1.24)	1.00(2)			7.50(3.62)	7.00(5)			2.77(1.45)	3.00(2)			1.14(1.46)	1.00(2)			1.74(1.48)	2.00(3)			1.32(1.56)	1.00(2)			6.97(3.97)	7.00(5)		
Definite need	162	3.36(1.38)	4.00(1)			1.47(1.48)	1.00(2)			1.68(1.51)	2.00(3)			1.32(1.38)	1.00(2)			7.83(4.03)	8.00(5)			2.81(1.43)	3.00(2)			1.23(1.17)	1.00(2)			1.78(1.60)	2.00(3)			1.55(1.49)	2.00(3)			7.38(4.17)	7.00(6)		
IOTN (AC) treatment need																																									
No need	469	3.45(1.32)	4.00(1)	0.153	0.160	1.23(1.37)	1.00(2)	0.514	0.272	1.51(1.51)	1.00(2)	0.265	0.295	1.01(1.21)	1.00(2)	0.003**	0.003**	7.20(3.66)	7.00(6)	0.092	0.060	2.72(1.38)	3.00(2)	0.208	0.167	1.14(1.21)	1.00(2)	0.602	0.599	1.61(1.45)	1.00(3)	0.349	0.339	1.43(1.54)	1.00(2)	0.556	0.440	6.90(3.83)	7.00(5)	0.161	0.187
Borderline need	89	3.57(1.47)	4.00(1)			1.39(1.28)	1.00(2)			1.76(1.57)	2.00(3)			1.39(1.25)	1.00(2)			8.12(3.60)	8.00(6)			2.98(1.53)	3.00(2)			1.27(1.34)	1.00(2)			1.85(1.50)	2.00(3)			1.61(1.48)	2.00(2)			7.71(4.06)	7.00(6)		
Definite need	31	3.03(1.28)	3.00(2)			1.39(1.31)	1.00(2)			1.35(1.33)	1.00(2)			1.55(1.55)	1.00(2)			7.32(3.65)	8.00(5)			2.55(1.31)	3.00(2)			1.06(1.32)	1.00(2)			1.65(1.43)	2.00(3)			1.35(1.31)	1.00(2)			6.61(3.33)	6.00(5)		
DAI																																									
Normal or minor malocclusion- no treatment need or slight need	312	3.41(1.32)	4.00(2)	0.875	0.836	1.13(1.28)	1.00(2)	0.060	0.074	1.43(1.47)	1.00(2)	0.093	0.139	0.92(1.08)	1.00(2)	0.000**	0.002**	6.89(3.49)	7.00(5)	0.009**	0.027*	2.64(1.36)	3.00(2)	0.147	0.203	1.11(1.20)	1.00(2)	0.701	0.700	1.53(1.40)	1.00(3)	0.115	0.163	1.35(1.46)	1.00(2)	0.342	0.366	6.63(3.70)	6.00(5)	0.063	0.083
Definite malocclusion -treatment selective	143	3.50(1.31)	4.00(1)			1.35(1.35)	1.00(2)			1.55(1.49)	1.00(2)			1.22(1.39)	1.00(2)			7.62(3.46)	7.00(5)			2.81(1.43)	3.00(2)			1.20(1.34)	1.00(2)			1.71(1.35)	2.00(3)			1.51(1.67)	1.00(2)			7.24(3.76)	7.00(5)		
Severe malocclusion -treatment highly desirable	87	3.51(1.41)	4.00(1)			1.45(1.46)	1.00(3)			1.90(1.69)	2.00(3)			1.17(1.20)	1.00(2)			8.02(4.04)	8.00(5)			3.00(1.46)	3.00(2)			1.26(1.24)	1.00(2)			1.80(1.68)	2.00(3)			1.67(1.64)	2.00(3)			7.74(4.30)	7.00(6)		
Very severe (handicapping) malocclusion-treatment mandatory	47	3.45(1.50)	3.00(1)			1.55(1.50)	1.00(2)			1.53(1.47)	1.00(2)			1.74(1.59)	2.00(3)			8.28(4.29)	8.00(6)			2.87(1.48)	3.00(2)			1.09(1.14)	1.00(2)			1.98(1.60)	2.00(3)			1.51(1.18)	2.00(2)			7.45(4.03)	8.00(6)		
ICON treatment need																																									
No	383	3.43(1.32)	4.00(1)	0.668	0.451	1.19(1.36)	1.00(2)	0.059	0.015*	1.50(1.52)	1.00(2)	0.442	0.348	1.01(1.17)	1.00(2)	0.031*	0.042*	7.13(3.68)	7.00(5)	0.053	0.019*	2.69(1.38)	3.00(2)	0.124	0.119	1.14(1.23)	1.00(2)	0.770	0.754	1.62(1.46)	1.00(3)	0.499	0.414	1.38(1.49)	1.00(2)	0.121	0.122	6.83(3.86)	7.00(5)	0.127	0.087
Yes	206	3.48(1.39)	4.00(1)			1.41(1.31)	1.00(2)			1.60(1.50)	1.00(3)			1.25(1.35)	1.00(2)			7.74(3.60)	8.00(5)			2.87(1.46)	3.00(2)			1.17(1.25)	1.00(2)			1.70(1.44)	2.00(3)			1.58(1.57)	1.00(2)			7.33(3.82)	7.00(5)		
ICON complexity																																									
Easy	173	3.42(1.31)	3.00(1)	0.546	0.482	1.10(1.31)	1.00(2)	0.433	0.262	1.48(1.51)	1.00(2)	0.822	0.812	0.98(1.17)	1.00(2)	0.077	0.108	6.98(3.74)	7.00(5)	0.429	0.295	2.69(1.35)	3.00(2)	0.091	0.085	1.16(1.29)	1.00(2)	0.634	0.736	1.44(1.40)	1.00(2)	0.213	0.220	1.42(1.48)	1.00(2)	0.886	0.755	6.71(3.94)	6.00(6)	0.216	0.221
Mild	292	3.47(1.30)	4.00(1)			1.32(1.40)	1.00(2)			1.53(1.53)	1.00(2)			1.06(1.25)	1.00(2)			7.37(3.60)	7.00(5)			2.72(1.43)	3.00(2)			1.15(1.17)	1.00(2)			1.73(1.47)	2.00(3)			1.44(1.59)	1.00(2)			7.03(3.82)	7.00(5)		
Moderate	67	3.63(1.55)	4.00(2)			1.33(1.33)	1.00(2)			1.73(1.62)	2.00(3)			1.27(1.23)	1.00(2)			7.96(3.98)	9.00(5)			3.19(1.36)	3.00(2)			1.33(1.45)	1.00(2)			1.82(1.57)	2.00(3)			1.63(1.43)	2.00(3)			7.97(4.01)	8.00(6)		
Difficult	33	3.30(1.49)	3.00(2)			1.39(1.20)	1.00(2)			1.58(1.23)	2.00(2)			1.30(1.29)	1.00(2)			7.58(3.08)	8.00(5)			2.67(1.47)	3.00(2)			1.03(1.10)	1.00(2)			1.58(1.25)	2.00(3)			1.39(1.52)	1.00(2)			6.67(3.36)	7.00(6)		
Very difficult	24	3.13(1.30)	3.00(2)			1.46(1.25)	1.00(2)			1.42(1.41)	1.00(3)			1.63(1.58)	1.50(3)			7.63(3.72)	7.50(4)			2.50(1.38)	2.50(3)			0.92(1.18)	0.50(2)			1.83(1.47)	2.00(3)			1.33(1.27)	1.00(2)			6.58(3.43)	7.00(5)		
PAR																																									
Almost ideal occlusion	122	3.43(1.40)	4.00(1)	0.963	0.993	1.07(1.21)	1.00(2)	0.025*	0.007**	1.45(1.39)	1.00(2)	0.671	0.892	0.99(1.18)	1.00(2)	0.134	0.264	6.94(3.63)	7.00(5)	0.229	0.194	2.66(1.42)	3.00(2)	0.236	0.230	1.16(1.12)	1.00(2)	0.887	0.853	1.51(1.42)	1.00(2)	0.481	0.471	1.34(1.54)	1.00(2)	0.691	0.576	6.68(3.76)	6.50(5)	0.547	0.608
Acceptable occlusion	254	3.46(1.25)	4.00(1)			1.20(1.41)	1.00(2)			1.59(1.61)	1.00(3)			1.03(1.17)	1.00(2)			7.29(3.69)	7.00(6)			2.69(1.37)	3.00(2)			1.18(1.32)	1.00(2)			1.70(1.45)	1.00(3)			1.48(1.52)	1.00(3)			7.04(3.81)	7.00(5)		
Malocclusion	213	3.44(1.43)	4.00(1)			1.46(1.33)	1.00(2)			1.52(1.47)	1.00(2)			1.23(1.36)	1.00(2)			7.64(3.64)	7.00(5)			2.88(1.44)	3.00(2)			1.12(1.20)	1.00(2)			1.67(1.48)	2.00(3)			1.48(1.52)	1.00(2)			7.15(3.96)	7.00(6)		

P1: *p* value of parametric tests; P2: *p* value of nonparametric tests; *: *p* < 0.05, **p < 0.01

Parametric tests: comparison between two samples used the independent samples t test; others used the one-way ANOVA; nonparametric tests: comparison between two samples used the Mann-Whitney U test; others used the Kruskal-Wallis H test

SiC *Index* Significant Caries Index

Mother’s education was linked more closely with children’s CPQ scores than father’s education was. Mother’s education had effects on all CPQ domains (ISF:8 or RSF:8). The higher the education level, the lower the scores. Father’s education only showed a significant effect on OS score. Household income showed no effect on CPQ scores.

Subjects with unhealthy periodontal conditions and high caries experiences had higher scores in all domains (ISF:8 and RSF:8). However, significant results only existed in the domains of EWB, SWB and total CPQ: periodontal status affected EWB and total CPQ (ISF:8), while caries affected SWB and total CPQ (RSF:8).

Three tendencies are shown by the descriptive statistics of malocclusion and CPQ scores. First, malocclusion measured by ICON treatment need showed that in all domains subjects with malocclusion had higher scores than those without malocclusion (ISF:8 and RSF:8). Second, malocclusion measured by PAR and IOTN (DHC) showed that a severer malocclusion was associated with a higher score in almost all domains, except that in OS domain, the RSF questionnaire had this tendency while the ISF did not. Third, in the domains of FL and SWB (ISF:8), all orthodontic indices showed that the severer the malocclusion, the higher the scores.

Different orthodontic indices generated different statistical results, of which ICON detected the most significant results. The significant results mainly existed in the domains of FL, SWB and total CPQ (Table 2).

In ordinal regression, the dependent variables were set as CPQ ranks (Table 3); higher ranks represented poorer quality of life. For gender, ordinal regression generated the same result with the bivariate analysis: compared with females, males tended to rank higher in OS domain but lower in EWB domain (adjusted OR = 1.89 and 0.67, respectively).

**Table 3:** Ordinal regression of associations between the factors and the CPQ_11–14_ scores

	OS (ISF:8)		FL (ISF:8)		EWB (ISF:8)		SWB (ISF:8)		CPQ_11–14_ ISF: 8 total score		OS (RSF:8)		FL (RSF:8)		EWB (RSF:8)		SWB (RSF:8)		CPQ_11–14_ RSF: 8 total score	
	OR (95%CI)	P	OR (95%CI)	P	OR (95%CI)	P	OR (95%CI)	P	OR (95%CI)	P	OR (95%CI)	P	OR (95%CI)	P	OR (95%CI)	P	OR (95%CI)	P	OR (95%CI)	P
Sociodemographic status																				
Gender																				
F^a^																				
M	1.89 (1.38, 2.59)	0.000**	1.13 (0.84, 1.53)	0.414	0.72 (0.53, 0.97)	0.030*	0.94 (0.70, 1.28)	0.702	1.14 (0.85, 1.54)	0.383	1.23 (0.91, 1.67)	0.180	0.77 (0.57, 1.04)	0.091	0.67 (0.49, 0.90)	0.008**	1.01 (0.75, 1.37)	0.928	0.96 (0.71, 1.29)	0.774
Father’s education																				
Primary school graduate or below^a^																				
Secondary school graduate or below	1.71 (1.04, 2.82)	0.035*	1.14 (0.71, 1.82)	0.595	2.10 (1.30, 3.40)	0.003**	1.26 (0.78, 2.04)	0.335	1.78 (1.11, 2.86)	0.017**	1.78 (1.10, 2.91)	0.020*	0.96 (0.60, 1.53)	0.863	1.70 (1.05, 2.73)	0.029*	1.41 (0.88, 2.27)	0.154	1.60 (1.00, 2.55)	0.0504
College graduate or above	1.12 (0.56, 2.26)	0.745	1.22 (0.63, 2.36)	0.554	1.96 (1.00, 3.81)	0.048*	1.60 (0.82, 3.12)	0.168	1.74 (0.90, 3.36)	0.100	1.51 (0.76, 2.98)	0.235	0.70 (0.36, 1.36)	0.293	2.62 (1.34, 5.09)	0.005**	1.50 (0.77, 2.92)	0.229	1.72 (0.89, 3.32)	0.105
Mother’s education																				
Primary school graduate or below^a^																				
Secondary school graduate or below	0.50 (0.30, 0.83)	0.007**	0.60 (0.37, 0.97)	0.039*	0.43 (0.26, 0.71)	0.001**	0.74 (0.45, 1.21)	0.224	0.49 (0.30, 0.80)	0.004**	0.41 (0.25, 0.68)	0.000**	0.61 (0.38, 1.00)	0.049*	0.52 (0.32, 0.85)	0.010*	0.53 (0.33, 0.87)	0.012*	0.45 (0.27, 0.73)	0.001**
College graduate or above	0.49 (0.23, 1.05)	0.066	0.50 (0.24, 1.03)	0.061	0.50 (0.24, 1.03)	0.061	0.71 (0.34, 1.48)	0.366	0.47 (0.23, 0.97)	0.040*	0.26 (0.12, 0.56)	0.000**	0.57 (0.28, 1.18)	0.132	0.64 (0.31, 1.33)	0.229	0.65 (0.32, 1.35)	0.249	0.37 (0.18, 0.76)	0.007**
Household income																				
Below HK$10,000^a^																				
HK$10,001-HK$20,000	1.28 (0.85, 1.93)	0.244	1.15 (0.77, 1.71)	0.490	1.24 (0.83, 1.84)	0.291	0.96 (0.65, 1.43)	0.853	1.09 (0.74, 1.62)	0.651	1.38 (0.92, 2.07)	0.115	1.00 (0.68, 1.48)	0.994	1.08 (0.72, 1.60)	0.715	0.98 (0.66, 1.45)	0.901	1.16 (0.78, 1.71)	0.464
HK$20,001-HK$30,000	0.84 (0.50, 1.42)	0.517	1.12 (0.68, 1.85)	0.646	1.13 (0.69, 1.85)	0.640	0.89 (0.54, 1.47)	0.643	0.85 (0.52, 1.40)	0.521	1.27 (0.77, 2.11)	0.351	0.91 (0.55, 1.49)	0.696	1.09 (0.66, 1.79)	0.737	0.72 (0.44, 1.19)	0.206	0.94 (0.58, 1.55)	0.821
HK$30,001-HK$40,000	1.50 (0.82, 2.73)	0.187	1.89 (1.06, 3.38)	0.031*	1.62 (0.90, 2.89)	0.106	0.87 (0.48, 1.57)	0.643	1.28 (0.72, 2.28)	0.400	1.51 (0.84, 2.73)	0.169	1.46 (0.82, 2.61)	0.197	1.12 (0.63, 2.01)	0.700	1.24 (0.70, 2.22)	0.465	1.49 (0.84, 2.65)	0.175
Over HK$40,001	0.96 (0.50, 1.83)	0.894	1.34 (0.73, 2.48)	0.345	1.04 (0.56, 1.91)	0.903	0.69 (0.37, 1.29)	0.250	0.82 (0.44, 1.50)	0.516	1.06 (0.56, 1.99)	0.862	1.02 (0.55, 1.90)	0.940	0.64 (0.34, 1.19)	0.155	0.77 (0.41, 1.42)	0.395	0.86 (0.47, 1.57)	0.615
Malocclusion																				
IOTN (DHC) treatment need																				
No need^a^																				
Borderline need	0.91 (0.60, 1.40)	0.681	1.30 (0.86, 1.94)	0.210	0.92 (0.61, 1.38)	0.681	1.07 (0.71, 1.61)	0.760	1.10 (0.73, 1.65)	0.645	1.12 (0.74, 1.69)	0.584	0.85 (0.56, 1.28)	0.424	1.12 (0.75, 1.69)	0.577	0.75 (0.50, 1.14)	0.179	0.87 (0.58, 1.30)	0.505
Definite need	0.86 (0.60, 1.23)	0.415	1.45 (1.03, 2.05)	0.034*	1.29 (0.92, 1.82)	0.145	1.52 (1.08, 2.16)	0.017*	1.33 (0.94, 1.87)	0.107	1.10 (0.77, 1.56)	0.596	1.24 (0.88, 1.75)	0.219	1.24 (0.88, 1.76)	0.220	1.16 (0.82, 1.64)	0.396	1.23 (0.87, 1.73)	0.234
IOTN (AC) treatment need																				
No need^a^																				
Borderline need	1.17 (0.76, 1.79)	0.477	1.30 (0.86, 1.97)	0.216	1.32 (0.87, 2.00)	0.189	1.90 (1.25, 2.88)	0.003**	1.54 (1.02, 2.33)	0.041*	1.45 (0.95, 2.20)	0.083	1.14 (0.75, 1.72)	0.549	1.31 (0.86, 2.00)	0.203	1.26 (0.83, 1.91)	0.283	1.48 (0.98, 2.23)	0.065
Definite need	0.58 (0.28, 1.21)	0.147	1.24 (0.64, 2.40)	0.519	0.90 (0.46, 1.75)	0.758	2.06 (1.07, 3.98)	0.031*	1.23 (0.64, 2.38)	0.532	0.76 (0.38, 1.51)	0.434	0.79 (0.41, 1.56)	0.504	1.03 (0.53, 2.00)	0.928	0.97 (0.50, 1.89)	0.928	0.91 (0.47, 1.77)	0.790
DAI severity and treatment need																				
Normal or minor malocclusion- no treatment need or slight need^a^																				
Definite malocclusion- treatment selective Definite malocclusion- treatment selective	1.14 (0.78, 1.67)	0.490	1.35 (0.94, 1.94)	0.109	1.25 (0.87, 1.79)	0.235	1.49 (1.03, 2.15)	0.035*	1.35 (0.94, 1.94)	0.102	1.28 (0.89, 1.86)	0.188	1.08 (0.75, 1.56)	0.671	1.44 (0.99, 2.07)	0.054	1.13 (0.78, 1.63)	0.508	1.37 (0.95, 1.96)	0.091
Severe malocclusion- treatment highly desirable	0.90 (0.57, 1.43)	0.660	1.34 (0.87, 2.08)	0.184	1.71 (1.10, 2.65)	0.016*	1.51 (0.97, 2.34)	0.068	1.44 (0.94, 2.23)	0.097	1.38 (0.89, 2.14)	0.154	1.29 (0.84, 2.00)	0.250	1.25 (0.81, 1.95)	0.311	1.37 (0.88, 2.12)	0.161	1.59 (1.03, 2.45)	0.037*
Very severe (handicapping) malocclusion-treatment mandatory	0.90 (0.50, 1.63)	0.736	1.75 (1.00, 3.05)	0.0499*	1.16 (0.67, 2.04)	0.594	3.06 (1.74, 5.36)	0.000**	1.90 (1.09, 3.33)	0.024*	1.19 (0.67, 2.11)	0.547	1.06 (0.60, 1.86)	0.840	1.81 (1.03, 3.19)	0.041*	1.39 (0.80, 2.44)	0.246	1.64 (0.94, 2.87)	0.081
ICON treatment need																				
No^a^																				
Yes	1.15 (0.83, 1.59)	0.408	1.39 (1.02, 1.90)	0.040*	1.13 (0.83, 1.55)	0.432	1.36 (1.00, 1.87)	0.053	1.40 (1.02, 1.91)	0.035*	1.25 (0.91, 1.72)	0.165	1.05 (0.77, 1.44)	0.764	1.06 (0.77, 1.45)	0.715	1.20 (0.88, 1.65)	0.246	1.27 (0.93, 1.74)	0.126
ICON complexity																				
Easy^a^																				
Mild	1.07 (0.74, 1.53)	0.726	1.31 (0.92, 1.85)	0.132	1.03 (0.73, 1.45)	0.869	1.07 (0.76, 1.52)	0.690	1.13 (0.80, 1.59)	0.503	0.95 (0.67, 1.35)	0.783	1.03 (0.73, 1.46)	0.852	1.44 (1.01, 2.03)	0.041*	0.98 (0.69, 1.38)	0.895	1.14 (0.81, 1.61)	0.444
Moderate	1.54 (0.90, 2.61)	0.112	1.40 (0.84, 2.35)	0.198	1.28 (0.76, 2.13)	0.354	1.61 (0.96, 2.71)	0.070	1.48 (0.88, 2.46)	0.136	1.79 (1.07, 3.02)	0.027*	1.20 (0.72, 2.01)	0.486	1.52 (0.90, 2.55)	0.115	1.35 (0.80, 2.26)	0.257	2.01 (1.20, 3.37)	0.008**
Difficult	0.90 (0.44, 1.84)	0.771	1.54 (0.78, 3.04)	0.216	1.32 (0.67, 2.60)	0.427	1.73 (0.87, 3.42)	0.117	1.63 (0.83, 3.20)	0.160	1.01 (0.50, 2.01)	0.986	0.84 (0.42, 1.67)	0.617	1.30 (0.65, 2.58)	0.457	0.86 (0.43, 1.71)	0.665	1.03 (0.52, 2.03)	0.934
Very difficult	0.74 (0.32, 1.71)	0.480	1.71 (0.79, 3.71)	0.173	0.92 (0.42, 2.00)	0.836	2.28 (1.05, 4.95)	0.036*	1.45 (0.67, 3.15)	0.341	0.77 (0.34, 1.73)	0.531	0.69 (0.31, 1.55)	0.371	1.61 (0.74, 3.52)	0.233	0.95 (0.43, 2.08)	0.897	1.11 (0.51, 2.40)	0.789
PAR																				
Almost ideal occlusion^a^																				
Acceptable occlusion	0.91 (0.60, 1.37)	0.646	1.01 (0.68, 1.51)	0.958	1.04 (0.70, 1.54)	0.858	1.02 (0.69, 1.53)	0.910	1.07 (0.73, 1.59)	0.719	0.94 (0.63, 1.41)	0.762	0.86 (0.58, 1.28)	0.465	1.18 (0.79, 1.75)	0.422	1.22 (0.82, 1.82)	0.321	1.09 (0.74, 1.61)	0.662
Malocclusion	0.91 (0.59, 1.39)	0.657	1.57 (1.04, 2.37)	0.031*	1.01 (0.67, 1.52)	0.958	1.32 (0.87, 1.99)	0.192	1.31 (0.87, 1.97)	0.192	1.24 (0.82, 1.88)	0.316	0.86 (0.57, 1.30)	0.481	1.16 (0.77, 1.75)	0.471	1.19 (0.79, 1.80)	0.403	1.16 (0.77, 1.74)	0.480
Periodontal and carries status																				
Periodontal status																				
CPI score = 0^a^																				
CPI score > 0	1.44 (0.91, 2.30)	0.122	1.43 (0.92, 2.22)	0.113	1.61 (1.04, 2.49)	0.034*	1.43 (0.92, 2.24)	0.115	1.63 (1.05, 2.53)	0.028*	1.10 (0.71, 1.72)	0.665	1.19 (0.77, 1.84)	0.435	1.40 (0.91, 2.17)	0.127	1.12 (0.73, 1.73)	0.605	1.18 (0.76, 1.81)	0.462
Caries experience																				
< SiC Index value^a^																				
> =SiC Index value	1.06 (0.69, 1.63)	0.799	1.33 (0.88, 2.01)	0.175	1.18 (0.78, 1.79)	0.426	1.31 (0.87, 1.99)	0.197	1.29 (0.85, 1.94)	0.227	1.16 (0.76, 1.76)	0.491	1.08 (0.72, 1.64)	0.702	1.36 (0.89, 2.06)	0.153	1.60 (1.06, 2.42)	0.026*	1.34 (0.89, 2.02)	0.165

Statistical method: Ordinal regression (link function: logit), each orthodontic index adopted one separate ordinal regression; dependent variable: CPQ scores classified into four groups with cut-off points as quartile (1: scores < =first quartile; 2: first quartile < scores < =second quartile; 3: second quartile < scores < = third quartile; 4: scores > third quartile); a: reference group; OR: adjusted odds ratio; CI: confidence interval; *: *P* < 0.05. **: *P* < 0.01

N: sample size; adjusted OR: malocclusions adjusted for gender, father’s education level (primary school graduate or below; secondary school, post-secondary or above), mother’s education level (levels set as father’s education), household income (Below HK$10000, HK$10001-HK$20000, HK$20001-HK$30000, HK$30001-HK$40000, HK$40001 or above), caries experience (DMFT < SiC Index value, DMFT > =SiC Index value), and periodontal status (CPI score = 0, CPI score > 0); gender, socioeconomic status, periodontal and caries status adjusted for the previous variables and malocclusion measured by DAI

*OS* Oral symptoms domain, *FL* Functional limitations domain, *EWB* Emotional well-being domain, *SWB* Social well-being domain, SiC *Index* Significant Caries Index

Results of statistical analysis

Mother’s education had a positive effect on children’s CPQ rank; while father’s education had almost a reverse effect (Table 3). Take total CPQ for example: compared with the lowest education level, the adjusted ORs for mother’s middle and highest levels of education were respectively 0.45 and 0.37 (*P* = 0.001 and 0.007) (RSF:8); whereas the adjusted OR for father’s middle level of education was 1.78 (*P* = 0.017) (ISF:8). Multivariate analysis detected more significant results than bivariate analysis did, for it detected an effect of father’s education not only on OS, but also on EWB and total CPQ after adjusting other factors.

Household income did not show significant results in all domains, except that in FL domain, compared with the lowest income, the “HK$30,001-HK$40,000” group was associated with 1.89 times the odds of having a higher FL rank (*p* = 0.031) (ISF:8); while no effect was detected by bivariate analysis.

Unhealthy periodontal conditions had a negative effect on EWB and CPQ ranks (adjusted OR = 1.61 and 1.63, respectively) (ISF:8), which was the same with the result of bivariate analysis; high caries experience only had a significant effect on SWB rank (adjusted OR = 1.60) (RSF:8), but not on total CPQ.

Malocclusion mainly affected FL, EWB, SWB and total CPQ, of which SWB was the most affected domain. This was the same with the result of the bivariate analysis. Different orthodontic indices generated different results, of which DAI detected the most significant results.

Generally speaking, a severer malocclusion was associated with a higher likelihood of having a higher rank. However, statistical results showed that only in SWB, all malocclusion severities measured by IOTN (AC) had a significant effect; in other CPQ domains, only the severe and/or the very severe malocclusion showed significant effects. Take the total CPQ for example: when compared with the no/minor malocclusion measured by DAI, only the severe and the very severe malocclusion were associated with high likelihoods of having a higher CPQ rank after adjusting the effects of other factors (adjusted OR = 1.59 and 1.90, respectively) (ISF:8 or RSF:8).

## Discussion

This research was a cross-sectional analysis on the influence factors of OHRQoL. Males tended to have worse OS but better EWB. Mother’s education had more effect on children’s CPQ scores than father’s education did; higher levels of mother’s education were associated with lower CPQ scores of their children, whereas the effect of father’s education was opposite. Household income showed little effect on OHRQoL. Unhealthy periodontal conditions had a worse effect on EWB and CPQ, while high caries experience had a worse effect on SWB. Malocclusion could affect FL, EWB, SWB and total CPQ, of which SWB was the most affected domain. All malocclusion severities had a worse effect on SWB, but only severe malocclusions had an effect on other domains.

Males experienced worse OS but higher EWB. It may reveal that males were more tolerant of oral symptoms than females were. It was mother’s education, but not father’s education or household income, that had a positive effect on children’s OHRQoL. Parents shoulder different responsibilities in a family unit; under most circumstances, mother is the main caregiver of children. Caregivers with high education levels may have more sense of children’s oral hygiene and health, thus their children tend to have better OHRQoL.

Unhealthy Periodontal conditions were more prevalent than caries in this 12-year-old cohort. There may be two possible reasons: first, caries has effective preventions like water fluoridation; second, in puberty period children are more susceptible to gingivitis [31].

Periodontal conditions could affect EWB and the total CPQ. Children with unhealthy periodontal conditions may feel upset, irritable or frustrated because of their teeth. High caries experience only had an effect on SWB, but not on OS or FL as in common knowledge. Hong Kong is an economically developed area, where government puts great efforts on preventive and educational measures of children’s oral health. Therefore, children’s teeth of this 12-year-cohort were normally in good condition; even if there was caries, the erosions were almost either shallow ones or had been well treated, which tended not to cause pulpal sensibility and pain.

SWB was the most detected domain affected by malocclusion. Children with malocclusion were more likely to be teased or called names; they might avoid smiling or speaking loud in class, and they argued more frequently with other children or with their family because of their teeth.

In this study, the effects of the severe and very severe malocclusions on the domains of FL, EWB and total CPQ were detected. Studies have shown that severe malocclusion could add difficulties of plaque cleaning, which cause periodontitis; plus, temporomandibular disorders are more likely to occur in subjects with severer malocclusion than in those with less severe or no malocclusion [32, 33]. Therefore, children with severe malocclusion may have higher possibilities of having function limitations and emotional burdens.

Orthodontic indices put emphasis on different malocclusion traits and generated different results. Take IOTN (AC) for example: this index only reflects the frontal traits of dental arches, in other words, the frontal aesthetics of subjects. No inter- or intra- arch malocclusion is considered [25]. Dental aesthetics usually affect people’s social attractiveness; thus IOTN (AC) easily detected the significant result in SWB. Therefore, this index may be perfect to judge the extent of the effect of malocclusion on subjects’ social lives. The higher the IOTN (AC) rates, the worse the subjects’ social well-beings.

ICON adopts IOTN (AC) for its aesthetic judgment and puts a great weight on it. At the same time, some other malocclusion traits, such as crowding and inter-arch relationship, are also assessed [20]. DAI also puts great weights on frontal aesthetics, and the inter-arch malocclusion is also considered; literatures showed that this index is particularly sensitive to occlusal conditions causing psychological or social dysfunctions [22]. Hence, ICON and DAI could easily detect the effect of malocclusion on the domain of SWB, and on other domains.

PAR measures all occlusal anomalies based on experts’ judgment of their deviation from normal occlusion [21]. In this research, CPQ scores showed a gradient ascent across PAR rates. IOTN (DHC) is based on the criteria drawn up by the orthodontic section of the Swedish Dental Society and the Swedish Medical Board (1966), which is also the authoritative judgments of occlusal anomalies [34]. It showed the same gradient ascent with PAR. Both indices could easily examine the effect of malocclusion on FL; IOTN (DHC) further detected the effect on SWB.

The sociodemographic and clinical factors that may influence OHRQoL were analyzed in this article based on a population-based sample. Given its cross-sectional analysis, the results should be treated with caution. The sample of this research was selected in Hong Kong. When generalizing the conclusion to other regions, the differences of geographical, cultural, and economical factors also need to be considered. Subjects in this research would be followed up in their 15- and 18- years old. The results of longitudinal observations should provide more definitive evidences.

## Conclusion

The influence factors of OHRQoL in a representative sample of 12-year-old children were studied. Males were more tolerant of oral symptoms than females were. Mother’s education level was more positively associated with children’s OHRQoL than father’s education was. Household income had little effect on OHRQoL. Unhealthy periodontal conditions could result in a worse emotional well-being; while high caries experience could result in a worse social well-being. All malocclusion severities affected social well-being, while severe malocclusions further led to functional limitations, worse emotional experiences, and hence worse OHRQoL.

## Abbreviations

AC: aesthetic component
CPI: Community Periodontal Index
CPQ: child perceptions questionnaire
DAI: dental aesthetic index
DHC: dental health component
DMFT: Decayed, Missing and Filled Teeth
EWB: emotional well-being
FL: functional limitations domain
ICON: index of complexity, outcome and need
IOTN: index of orthodontic treatment need
OHRQoL: oral health-related quality of life
OR: odds ratio
OS: oral symptoms domain
PAR: peer assessment rating
SD: standard deviation
SE: standard error
SiC index: Significant Caries Index
SWB: social well-being
WHO: World Health Organization
